# Edible Insects as Food–Insect Welfare and Ethical Aspects from a Consumer Perspective

**DOI:** 10.3390/insects13020121

**Published:** 2022-01-25

**Authors:** Nora Delvendahl, Birgit A. Rumpold, Nina Langen

**Affiliations:** Department Education for Sustainable Nutrition and Food Science, Technical University Berlin, 10587 Berlin, Germany; n.delvendahl@tu-berlin.de (N.D.); nina.langen@tu-berlin.de (N.L.)

**Keywords:** edible insects, ethical aspects, insect welfare, consumer acceptance, consumer perception

## Abstract

**Simple Summary:**

For prevalent livestock, animal welfare is important to consumers. With increasing interest in edible insects, one might wonder how this concern translates to consumers’ perceptions of the welfare of insects. Therefore, we focus on consumers’ acceptance of how edible insects are currently produced. We first define what animal welfare means for prevalent livestock and transfer relevant aspects to the welfare of insects. Then, we review relevant aspects that shape consumers’ understanding of animal welfare. We provide an overview of the few consumer studies on insect welfare. Last, we present the public discourse on insects and discuss how this might be relevant to consumers’ perceptions of insect welfare.

**Abstract:**

A growing number of studies underline consumers’ concerns about the importance of animal welfare as a general concept for consumers’ purchase decisions. In particular, consumers perceive animal husbandry to be one of the most important aspects of animal welfare. Since intensive livestock production is criticized across society, the acceptance of current intensive production systems of edible insects is an issue of investigation. Criteria of insect welfare might differ from vertebrate welfare. One might argue that it is difficult to define standards for insect welfare due to their large diversity in living environments and feed requirements. In addition, it is debated whether insects are conscious and suffer from pain. It has been demanded to rear insects preferably under natural living conditions and some researchers proposed to consider them as sentient beings. Basic welfare and ethical aspects of insects as food and feed include species-specific mass rearing conditions and euthanasia, i.e., killing procedures. Consumers’ opinions and concerns regarding this issue have hardly been considered so far. In this paper, the animal welfare of prevalent livestock is defined and outlined, and relevant criteria are transferred to insect welfare. Different ways consumers might arrive at an animal welfare understanding are discussed, along with an overview of the few consumer studies on insect welfare. Furthermore, we consider how insects are presented in the public discourse and infer how this might be relevant to consumers’ perceptions of insect welfare.

## 1. Introduction

The consumption of insects as food, referred to as entomophagy, is traditionally practised in many cultures [1] with approximately several hundreds of million people worldwide regularly eating insects [2]. In recent years, edible insects have been widely discussed as a sustainable and economic alternative for other sources of animal protein, such as meat from conventional livestock [3,4]. Food made of (or based on) insects is also growing in popularity in the Western world [5] but from a low consumer acceptance level in Western societies [6]. To make insect protein available throughout the world requires large-scale sustainable insect production on an industrial level. Paving the way for the edible insect sector to expand on the European market, the European Food Safety Authority (EFSA) recently assessed the dried yellow mealworm (*Tenebrio molitor* larvae) [7] and frozen and dried migratory locusts (*Locusta migratoria*) [8] as safe for human consumption (see also: Commission Implementing Regulation (EU) 2021/882) [7]. Underlining this trend, the insect-farming business is predicted to increase tenfold globally by 2025 [9]. As more insect-based products enter the Western market, it seems necessary to assume that more questions will be raised regarding their production (e.g., see [10]). With regard to prevalent livestock production, several studies have highlighted consumers’ concern about and the importance of animal welfare as a general concept for consumers’ purchase decisions [11]. A comparison of two EU surveys, for example, highlights an increase in the importance people place on animal welfare [12,13]. Alonso et al. [14] show that public concern about animal welfare can be traced back to the nineteenth century with increasing relevance in the last years. What remains unclear, however, is whether and how exactly the animal welfare of farmed insects (also called mini-livestock) is perceived by consumers.

There is no universally-accepted definition of animal welfare with different stakeholders focusing on different aspects of this multidimensional concept [15,16,17]. Franz et al. [16] differentiate between three main approaches: (1) the “Biological Functioning Approach” focuses on animals being healthy, showing good productivity (e.g., daily growth) and reproducing well, (2) the “Affective States Approach” evaluates feelings and defines animal welfare as maximizing positive and minimizing negative feeling (e.g., suffering or pain), (3) the “Natural Living Approach” focuses on animals being able to express their “species-specific innate and natural behaviour” [16] (p. 1447). Focusing on consumers’ perception of animal welfare, Klink-Lehmann and Langen [11] found the most important aspect in which consumers are interested to be animal husbandry. Further underlining that husbandry practices are a key component of perceived animal welfare, intensive livestock production is widely criticized across society [13,18,19,20] and hardly a month passes without “livestock farming being publicly pilloried” [21].

Over the last years, several papers have raised the issue of animal welfare in the context of insect farming (e.g., [10,22,23,24,25,26]). In addition, the International Platform of Insects for Food and Feed [27] has been trying to promote insect welfare by applying Brambell’s [28] Five Freedoms to insect farming and encouraging producers to follow them. Similarly, the Dutch Animal Act (see [23]), which is based on the Five Freedoms includes some insect species as “production animals”. However, except for the Finnish Food Safety Authority [29], which specifically mentions animal welfare regulations for insects, the Thai National Bureau of Agricultural Commodity and Food Standards [30] and FAO [31], which both published cricket farming guidelines, and the Dutch Animal Act (see [23]), which lists some insect species as ‘production animals’, there are currently few official species-specific regulations regarding insect welfare. While insects fall under the category of “farmed animals” according to EU regulations and there are health and sanitation regulations regarding their production (see Lotta, 2019 for a discussion of the legal framework for insects as food), invertebrate animals are not included in the EU’s animal welfare directive [32]. With regard to Germany, the animal protection law (Tierschutzgesetz) holds for all animals, including invertebrates, but most specifications, such as the need for pain avoidance and/or anesthesia when it comes to slaughtering, only regard vertebrates [33].

Based on this lack of welfare regulations for insect farming, this paper aims to review existing literature on insect welfare and focuses on the acceptance of current production systems of edible insects. First, the animal welfare of conventional livestock is defined and outlined. Based on that, we will identify relevant aspects to consider for insect welfare. We will contemplate different ways consumers might arrive at an animal welfare understanding and how this could link to their views of insects. Furthermore, an overview of the few existing consumer studies on insect welfare is given and related to the insights regarding animal welfare. Last, it is discussed how insects are presented in the public discourse and inferred how this might be relevant to consumers’ perceptions of insect welfare.

## 2. Overview of Animal Welfare for Prevalent Livestock

One approach to animal welfare that has been developed for the welfare of intensively farmed livestock is Brambell’s Five Freedoms [22,23,27,34,35]. These state that all farmed animals should have: (1) freedom from hunger and thirst, (2) freedom from discomfort, (3) freedom from pain, injury, or disease, (4) freedom to express normal behavior, (5) freedom from fear and distress (see Table 1). Combining the Biological Functioning, the Affective States and the Natural Living Approach, the Five Freedoms have been widely influential to animal welfare legislations [34]. The European Commission’s animal welfare regulations (see, e.g., Council Directive 98/58/EC, Council Regulation (EC) No 1/2005, Council Regulation (EC) No 1099/2009), for example, are designed to reflect the Five Freedoms. Their legislation lays out specific welfare requirements for different livestock species regarding animal husbandry practices on the farm, during transport, and at slaughter [14]. On the farm, some aspects to consider are housing systems (i.e., space, structuring, light, temperature), potentially painful interventions, and/or vet procedures and feed (see Council Directive 98/58/EC). Based on this, the European Commission defined minimum standards, which are species-specific as different species have different welfare requirements and issues (e.g., tail docking for pigs (Council Directive 2008/120/EC) vs. tethering for calves (Council Directive 2008/119/EC). Using space in housing systems as an illustrative example, pigs (specifically weaner or rearing pigs) kept in a group are required to have at least between 0.15–1 m^2^ of usable space per animal depending on the weight (Council Directive 2008/120/EC), whereas for laying hens in enriched cages it is a minimum of 750 cm^2^ (i.e., 0.075 m^2^) per animal (Council Directive 1999/74/EC). It should be noted, of course, that these minimum standards are heavily criticised (e.g., in the context of intensive livestock farming) [13,18,19,20,21]. Stricter regulations are employed by specific animal welfare certifiers (e.g., Neuland) or sometimes organic certifiers (e.g., Naturland) who lay out species-specific welfare requirements that are above the legal minimum standard.

Consumers commonly seem to equate animal welfare with ‘animal husbandry’ while simultaneously neglecting aspects such as transport or slaughtering [11,36]. In line with this, De Jonge & van Trijp [19] found that for broiler chickens, outdoor access, stocking density, and day-night rhythm (i.e., animal husbandry practices) were judged as particularly influential to animal friendliness, whereas other practices such as transport duration or the type of breed were perceived to have less of an impact. Interestingly, individual differences in perception were linked to knowledge and familiarity with livestock farming (as well as moral beliefs and degree of anthropomorphism). This may be due to a lack of a universally accepted definition of animal welfare as well as the increasing distance between consumers and livestock production [12,37,38].

Despite this growing concern and the importance of animal welfare as a general concept for consumers’ purchase decisions, the market share for animal welfare certified products remains relatively small [11]. One reason for this attitude-behaviour gap appears to be social desirability bias (i.e., the tendency of individuals to respond in a way that they believe is socially acceptable) [14,36]. Furthermore, consumers might not feel responsible for animal welfare [18]. Indeed, most people seem to believe that animal welfare should be either regulated by public authorities or jointly handled by businesses and public authorities [13,18]. Parallel to this, consumers are commonly only willing to pay a small amount of money extra for more animal friendly products, as illustrated by the 2016 Eurobarometer survey, 35% of respondents reported that they would not be ready to pay more for animal friendly products and a further 35% would not be ready to pay over 5% more [13]. Looking into different animal welfare labelling and certification schemes, Verbeke [15] remarked that “animal welfare per se has a relatively low potential for differentiation unless it can be linked and associated with other, more tangible, product qualities” (p. 327). From this perspective, a more efficient way of motivating consumers to purchase animal friendly products might be to integrate animal welfare standards that are above the legal minimum into the general idea of quality [15].

## 3. Aspects concerning the Animal Welfare of Insects

While regulations for the animal welfare of conventional livestock are clearly defined, there are few official standards for insect farming [32]. Several guidelines regarding the production of different insect species exist [31,39,40]. However, these are not binding regulations, which means that insect farmers work in a “legislative grey zone” [22] (p. 238) and have to actively seek out information. In addition, few guidelines specifically mention animal welfare as an aspect to consider when keeping insects. Consequently, insect farmers often have to guess and ‘trial-and-error’ best practices [22,25,26]. Interviewing twelve insect farmers and one representative of the insect industry in the UK, Bear [25,26] exemplifies how producers perceive and cope with the “ethical ambiguity of insect production” [26] (p. 1011). Regarding acceptable slaughter methods, for example, one insect farmer reported that they had to “make it up” [25] (p. 760), and another farmer stated: “I wouldn’t have thought being frozen stiff was especially nice, but I have no idea and it just seems to be a reasonable way to do it” [25] (p. 761). These qualitative findings point to a need for welfare regulations that clearly guide insect farmers through the different stages of insect production.

Based on differences in their biology, it appears plausible to assume that insect animal welfare might differ from vertebrate welfare. For example, even though specific space requirements vary between species, high stocking density is often associated with poorer animal welfare for prevalent livestock [34]. Based on this, specific regulations have been put in place to limit stocking density and allow for sufficient space per animal (see Council Directive 98/58/EC). For industrial insect farming, in contrast, high stocking density has been claimed to not be problematic because “many insect species naturally live in large groups in small amounts of space” [41] (p. 125). The optimum density of an insect farming container, however, needs to be considered on a species level as some species, such as the black soldier fly (*Hermetia illucens*) larvae, may overheat in high densities [23]. And with regard to the cricket *Gryllus bimaculatus*, population density has been shown to drastically influence behavioural, morphological, and physiological aspects with high population density being linked to suppressed growth and development as well as different behaviour, such as decreased aggressiveness, increased activity and response to tactile, visual or olfactory stimuli [42,43]. These examples highlight that insect welfare regulations need to be species-specific.

An increasingly popular approach for insect welfare is to apply the Five Freedoms for farmed animal welfare to insects. De Goede et al. [22] and more recently, the Finnish Food Safety Authority [29] and the International Platform of Insects for Food and Feed (IPIFF) [27] formulated suggestions for insect welfare based on these Five Freedoms. The benefit of this approach is that the Five Freedoms are a well-established set of principles that incorporate the different aspects of animal welfare [15,16,17].

Yet, a potential problem with the Five Freedoms is their general view on farmed animals without defining species-specific aspects. For aspects that significantly vary across species, the recommendations commonly suggest taking species-specific needs into account rather than explicitly listing or describing these. Taking the ‘freedom of discomfort’ as an example, the IPIFF [27] suggests to “respect the physiological needs of the insects, providing them with the most adequate environment”. This recommendation requires insect farmers to research these specific physiological needs for the species they are keeping. Given the large diversity in living environments and feed requirements of different insect species as well as uncertainties around what constitutes the ‘optimum’ environment for a specific species [26], this might pose potential problems for farmers wishing to adhere to these guidelines. Therefore, insect welfare standards should aim to provide tangible recommendations for specific species in the same way current regulations differentiate between different species (e.g., chicken vs. cow) for prevalent livestock.

Similarly vague, the ‘freedom from fear and distress’ raises the question of how to measure fear and distress in insects and whether insects are negatively affected by stress. So far, both De Goede et al. [22] and the IPIFF [27] point to future research instead of providing direct recommendations. Indeed, there does not appear to be a scientific consensus regarding stress responses in insects. Pali-Schöll and colleagues [23] suggested that “In line with what happens in mammals, hormone-mediated responses to stressful environmental conditions have been identified in some insects.” (p. 2765). It is important to note, however, that stress responses in insects might be species-specific [44,45,46]. Regarding honeybees, Even et al. [47] proposed a “hypothetical integrated honey bee stress pathway somewhat analogous to the mammalian HPA” (p. 1271). In crickets, high stocking densities result in smaller insects [43,48,49]. Furthermore, under starvation, the pupal stage starts earlier and takes longer for some insect species (e.g., *Bombyx mori*, *Psacothea hilaris*, and *Bactrocera dorsalis*) [46], suggesting that stress responses can negatively affect the insect yield. Of course, this does not provide information with regard to how insects subjectively experience environmental stressors. Thus, future research should aim to identify how and which insect species respond to different stressors.

Parallel to the ambiguities regarding insects’ stress response, the ‘freedom from pain, injury or disease’ links to the discussion of whether insects can experience pain. In the literature, a distinction is commonly made between ‘nociception’ (i.e., the capacity to respond to potentially damaging stimuli) and ‘pain’ (i.e., the subjective experience) [2,22,46]. While insects have been shown to have sensory neurons so-called nociceptors that respond to injury and damaging stimuli, this does not necessarily imply awareness of their nociceptive response [50,51]. In contrast to nociception, pain perception involves the subjective experience of “an aversive sensation and feeling associated with actual or potential tissue damage” [52] (p. 17). Thus, the experience of pain is linked to the ability to experience subjective states [50]. Given that insects cannot communicate verbally, investigating whether they subjectively experience pain is not straightforward. To overcome this, Sneddon and colleagues [53] developed a set of criteria to assess whether certain animal species experience pain, such as physiological and behavioural responses and motivational learning. To this point, however, there is no clear consensus whether insects have the capacity to feel, perceive or experience subjectively (i.e., whether they have sentience) [10,23,35,53,54].

**Figure 1:**
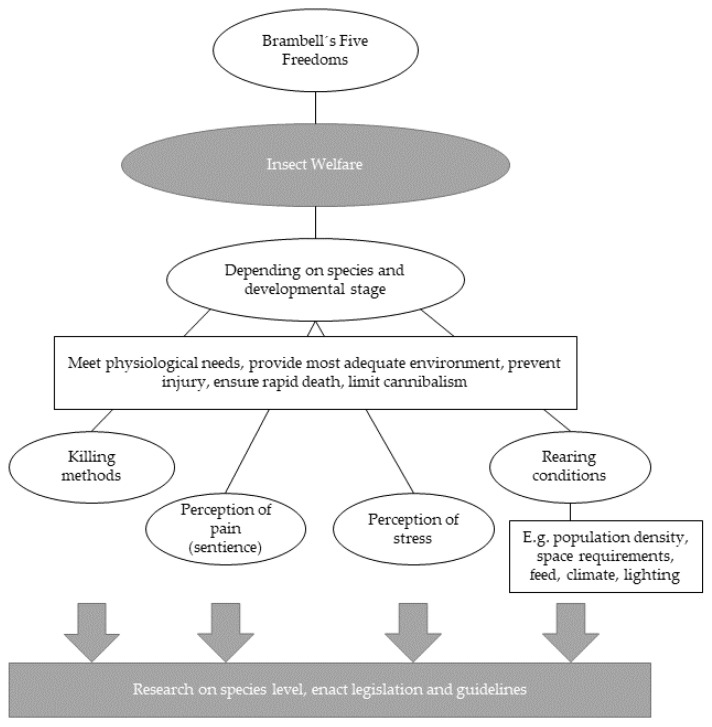
Summary of insect welfare aspects based on the Five Freedoms [27,28].

According to the ‘argument-by-analogy’, insects displaying behavioural and physiological responses that are similar to vertebrates are indicative of their pain perception [55]. Indeed, there are several studies showing examples of analogous behaviour [50,56]. *Drosophila* (both larvae and adult), for example, displays pain-like behaviour to noxious stimuli, such as heat or acid [57,58]. Furthermore, several insect species (i.e., cockroaches, *Drosophila*, ants, bees) engage in more self-grooming after injury and termite-hunting ants have even been found to treat other injured ants with the injured ants acting ‘more injured’ in the company of their nest-mates [56]. Regarding the subjective experience of pain, Gjerris et al. [35] argue that “absence of proof should not be misunderstood as proof of absence” [35] (p. 105). While until today we may not have definite proof of insects’ ability to have subjective experiences, this should not simply be taken as an argument against their ability. Indeed, from a neurological perspective, Klein and Barron [54] propose that “the insect brain supports functions analogous to those of the vertebrate midbrain and hence that insects may also have a capacity for subjective experience” (p. 1). Together with the evidence for insects’ pain-like behaviour, one might, therefore, reasonably suggest that insect farmers ought to act in line with the precautionary principle, which postulates that we should avoid any actions which are likely to cause pain in insects when this avoidance does not (or does only minimally) affect our own welfare [10,59]. Any actions that could cause harm to an insect should thus be avoided. In line with this, the IPIFF [27] recommends to “Prevent injury, ensure rapid death and limit cannibalism” and, therefore, opposes practices such as live-shredding of insects. Moreover, the EU organic regulations ban the mutilation of bees (e.g., clipping the wings of the queen bee, but do not consider other insect species (see Commission Regulation (EC) No 889/2008). Mandatory welfare regulations for edible insects are required that go beyond voluntary recommendations to ensure insect welfare, in parallel with further research on sentience and pain perception in insects (see Figure 1 for a summary of insect welfare aspects requiring further research and legislation).

One might argue that it is difficult to define standards for insect animal welfare due to their large diversity in living environments and feed requirements. Therefore, it might be more feasible to focus on the most commonly farmed insects for human consumption, such as crickets, grasshoppers, and mealworms [24]. Indeed, the German organic certifier Naturland is (to our knowledge) one of the pioneers regarding this as they provide separate animal husbandry regulations for *Coleoptera* (beetles), *Diptera* (flies), and *Saltatoria* (locusts) in addition to more general insect production regulations [60]. The general production regulations cover several welfare issues, such as cannibalism, housing systems, transport, mutilation, insect health, and slaughter, whereas the species-specific sections detail specific needs, such as feed, climate, lighting, and species-specific needs such as hiding places for locusts or stocking density for flies. As such, they provide comprehensive insect welfare recommendations for the species listed. It should be noted, however, that it is not quite clear how exactly these guidelines were derived. According to Naturland, their guidelines are based on various scientific papers and cooperation with experts, and they are in exchange with different organisations such as IPIFF (personal communication). Given the ambiguity of the exact rationale and basis of these regulations, it is possible that the predominant animal welfare approach might be ‘Biological Functioning’. If a study shows that the rate of cannibalism in mealworms is independent of larval density [61], this does not necessarily consider how mealworms live naturally (i.e., ‘Natural Living’) or how they might subjectively experience (i.e., ‘Affective States’) different stocking densities. Advances in research on insect sentience might aid in formulating insect welfare guidelines that encompass the different approaches.

One important aspect the Naturland guidelines [60] take into account is the different stages of metamorphosis. The guidelines provide information concerning the insects’ requirements at each developmental stage. This is crucial to insect welfare regulations as insects require different rearing conditions based on their developmental stage [23] parallel to how prevalent livestock has different welfare needs depending on their developmental stage (e.g., piglets vs. adult pigs).

## 4. Aspects Affecting People’s Animal Welfare Understanding (and Its Impact on Insect Welfare)

There are several ways to arrive at an animal welfare understanding. In the so-called Western world, consumers commonly base their understanding of animal welfare on an anthropomorphic perspective [11]. Anthropomorphism is defined as “the tendency to assign human characteristics—including emotions and cognitions—to animals and to objects” [62] (p. 27). With regard to animal welfare, an anthropomorphic perspective means that consumers project their ideas of what welfare and quality of life means for humans onto animals [11]. In general, anthropomorphism has been shown to increase pro-animal attitudes and support for animal welfare [63,64] and has been linked to empathy and motivation to protect animals [58,61]. Thus, the more people project human characteristics onto animals, the more they are concerned about animal welfare.

Interestingly, anthropomorphism varies based on phylogenetic distance with the animal [65]. Therefore, animals that are closer to humans regarding their morphology and behaviour are more anthropomorphized [65]. This has important consequences for their welfare and conversation: people are more willing to pay for and, thus, more resources are given to the conservation of vertebrates and animals that are phylogenetically closer to humans than to invertebrates who are phylogenetically more distant [62,63,64]. In line with this, Westbury and Neumann [66] found higher skin-conductance responses and subjective empathetic ratings towards suffering animals, the smaller the phylogenetic distance suggesting that empathetic concern for animal welfare is higher for phylogenetically close animals than for distant ones. These findings are highly relevant to consumers’ perspective of insect welfare, as the phytogenetical distance of humans to insects is much larger than to most prevalent livestock (especially vertebrates). From this perspective, one would expect consumers to be less concerned for the welfare of edible insects than for most prevalent livestock.

Linked to anthropomorphism, assigning mental capacities (i.e., sentience) to animals significantly affects their welfare and moral status [65]. Specifically, living beings who are perceived to have more mental capacities are attributed higher moral standing [67]. Thus, people show more moral concern for animals whom they perceive as having mental states, such as the capacity to experience pain. Given that anthropomorphism includes the attribution of mental states and that phylogenetically more distant animals are less anthropomorphised, this is crucial for whether and how people show moral concern for edible insects. Indeed, animal sentience has long been an important argument for animal protection and welfare [68,69]. In one of the most recent examples in the media, the UK government formally recognised animals as “sentient beings” by law along with announcing several animal welfare measures, such as a ban on live exports (e.g., see [70]). Notably, however, “sentient animals” are defined as vertebrates [71] meaning that invertebrates are explicitly excluded from the list of sentient beings. De Mori and Normando [68] argue that—even though there is no scientific base for it—people (including scientists and consumers) subscribe to the anthropocentric *scala naturae*, a hierarchical concept that ranks animals with regard to their cognition and sentience. Its hierarchy is commonly based on the species’ similarity, familiarity, or emotional importance to humans. In line with this, compared to most vertebrates, people commonly perceive invertebrates as less sentient (e.g., [69,72]). When asked to rate the mental capacities of different animal species in a Finnish study, for example, participants attributed the lowest scores to shrimp (the only invertebrate species in the list) [72]. While phylogenetic distance and, consequently, low anthropomorphism are plausible explanations for these findings, it is possible that new scientific advances in the field of invertebrate sentience (including pain perception) and effective communication strategies of these findings could change public perceptions.

Another aspect that influences concern for animal welfare is motivation. Several studies have shown that the boundaries of moral concern can change depending on peoples’ motivations [73] (see also [74]). Piazza and Laughnan [73], for example, found that even though intelligence is vital to an animal’s moral standing, people ignore this factor when it is in their own interest. Specifically, meat-eaters suddenly failed to take intelligence information into account for animals they consume when judging an animal’s moral standing, while generally considering animal intelligence when determining moral standing. Thus, the assessment of an animals’ moral status is not only dependent on one’s perception of its mental capacities but also on one’s own motivation, an aspect that could potentially be used to increase moral concern. Indeed, providing consumers with animal welfare information increases both intention to purchase and willingness to pay for products with higher animal welfare [75]. Informing consumers about welfare standards of edible insects (e.g., through labels) might, thus, be a way to increase motivation and concern for insect welfare.

The number of studies directly investigating consumers’ perceptions of insect welfare is limited. In one study, Polish consumers were asked whether insects should be included in Western diets to overcome animal welfare issues [76]. The most chosen answer with 38.8% was “I don’t know” followed by “yes” (33.5%) and 27.8% of respondents choosing “no”. While this finding might suggest that consumers are unsure whether there are fewer welfare concerns for insects compared to prevalent livestock farming, it does not allow for a clear interpretation. Other plausible explanations for the finding could be that consumers are unsure about whether edible insects should be included in Western diets for reasons other than insect welfare, or there might be a lack of knowledge regarding animal welfare in general. Another study also focusing on Polish consumers, Kostecka [77], investigated consumer acceptance of insect-based food. While the questionnaire included questions on the importance of animal welfare in food production and the acceptance of insect-based food, there was no analysis of the link between those questions (e.g., whether people who place higher importance on animal welfare reported lower acceptance levels of insect-based food). In a Norwegian and a Portuguese sample, however, ecological welfare (as measured via the food choice questionnaire) did not significantly predict the likelihood of accepting insects as food [78]. This finding could imply that consumers do not necessarily link animal welfare with insect products. However, future studies are needed to explicitly examine consumers’ knowledge as well as their attitudes towards insect welfare.

Last, indirectly hinting at consumers’ potential interest in insect welfare, a UK insect farmer in Bear’s study [25] stated that “(t)he consumer would want to know that they’re [the insects] being killed still with, whether we know it or not, with the fact that they might have pain in mind, you know.” [25] (p. 760). While quantitative studies are needed to test whether this perspective holds true for larger groups of insect farmers, it does suggest that producers might sense a concern for insect welfare on the consumer side.

## 5. How Insects Are Presented in the Public Discourse

Consumers often lack knowledge of animal husbandry practices and few have personal experiences, which means that their perception of animal husbandry (and welfare) is commonly influenced by the media [37,69]. Miele [79], for example, reported that across focus groups in different EU countries, “participants seemed to derive their (indirect) knowledge mainly from the mass media, to a large extent fashioned by a scandal-driven media that focused predominantly on negative issues (e.g., Bovine spongiform encephalopathy (BSE), salmonella outbreaks, Foot-and-mouth disease (FMD), dioxin, etc.)” [79] (p. 3). Parallel to this, knowledge about entomophagy and farmed insects is also often reported to come from the media [80,81]. Thus, the way in which insects are perceived is likely to be shaped by media discourse. A recent media analysis focusing on Finnish media coverage of entomophagy found that insects are commonly not described as animals [82]. Instead, they are commonly “referred to as products, biomass, raw material, grocery, ingredient, mass and particles” [82] (p. 312). Remarkably, the killing of insects is called ‘harvesting’, a term otherwise employed for crops, such as cereal. Given that knowledge of edible insects and their production seems to be formed by the media, it seems plausible to assume that their frequent depiction as inanimate products might drive the way in which consumers perceive them.

Interestingly, categorising a novel animal as food appears to reduce concern over its welfare [74]. Bratanova et al. [74] showed that when participants were presented with a novel animal in a frame that refers to it as being eaten, including preparation methods and flavour as opposed to presenting it as an animal with information about its population size and reproduction rate decreased the animal’s perceived capacity to suffer, which lowered moral concern. Assuming that consumers may have limited knowledge of edible insect species prior to encountering information about them in the media, this further underlines how insects’ media portrayal could result in low moral concern for insects.

Parallel to insects not being described as animals [71], the term ‘entovegan diet’ suggests that the consumption of insects may be compatible with an otherwise plant-based diet [82]. As such, insects are not perceived as an immoral food source such as meat. Supporting this idea, a qualitative study investigating consumers’ perspective of edible insects found that insects are perceived as more “ethical” (referring to both sustainability and animal welfare) than conventional meat not only by meat-eaters but also vegetarians [83]. Thus, even though vegetarians reported concern for animal welfare, this concern seemed to exclude insects. Linked to this, participants did not necessarily regard animals as insects, a theme that emerged in the interviews with both meat-eaters and vegetarians. In line with this, Elorinne et al. [84]) found vegetarians—more so than omnivores—to hold positive attitudes towards entomophagy. It should be noted, however, that vegan participants did not follow the same pattern and considered entomophagy as immoral. Regarding the ethicalness of insect consumption, Santaoja and Niva [82] propose that it is assumed to be ethical by default, because of “the allegedly smaller environmental impact as a central argument for ethicalness” [82] (p. 311). From this perspective, consumers may not question the ethical standards of edible insect production, as the consumption of insects in itself is perceived as a sustainable choice. Thus, unless animal welfare issues of certain insect production methods are highlighted in the media, it is unlikely that consumers will display high levels of concern.

There also appears to be a difference in perception between different types of insects. Specifically, ‘good’ insects, such as butterflies or bees, are differentiated from other types of insects towards which many people feel disaffection [24]. Honey bees, in particular, have received extensive media coverage and are commonly portrayed positively. Analysing the media coverage of honey bee colony losses, Huber & Aichberger [85] reported that journalists used neologisms, such as “bee killer” or “bee disaster”, to describe the issue and humanised bees in their articles (e.g., “hard-working staff”, “Maja the bee is finally able to laugh again”; [85] (p. 145)). Unlike the media coverage on entomophagy, this portrayal implies that honey bees are living animals that can be killed (as opposed to ‘harvested’). Moreover, as anthropomorphism is linked to moral concern, humanising honey bees might evoke more concern for their welfare. Based on this, it would be interesting to examine whether humanising and positively portraying edible insect species along with mentioning current welfare issues could influence consumers’ perceptions.

Insects are often perceived as ‘alien’ and many species commonly evoke a disgust response in humans [86]. From a psychological perspective, disgust has been linked to dehumanisation and moral exclusion, which might be why people struggle to empathise with insects [86]. One way to potentially overcome these negative attitudes is experience. Indeed, experience and familiarity with animals has been shown to influence concern for animal welfare (see [69]). Regarding prevalent livestock, perceptions of farm animals and concern for their welfare is linked to direct experience [87,88]. Tawse [87] found that students who had visited a conventional working pig farm reported more concern for farm animal welfare in general and pig welfare in specific in addition to being more willing to pay for increased welfare products. Applying this finding to insect welfare, facilitating direct experiences with insect farms (e.g., through public events or, less directly, through media coverage) might increase awareness and concern for their welfare. Illustrating how direct experience can also shape perceptions of animal minds, Hazel and colleagues [88] found a practical class during which students trained chickens in clicker tasks positively influenced students’ attitudes towards and intelligence perception of chickens. Following on from this, future research could investigate whether similar effects could be achieved with different insect species.

## 6. Conclusions

With consumers’ increasing concern for animal welfare and the predicted rise of the insect-farming sector, binding insect welfare regulations are required that consider individual species and their different developmental stages. For each species, these regulations should aim to lay out specific aspects of insect farming (e.g., housing, feed, transport, killing), parallel to current welfare regulations for prevalent livestock. While drawing on literature from both animal welfare and human-animal relationships suggests that consumers might not readily be concerned with the welfare of edible insects, their perceptions might at least partially be shaped by the current public discourse. The current lack of consumers studies explicitly focusing on perceptions of insect welfare highlights the need to investigate consumers’ attitudes, knowledge, and behaviour regarding insect welfare and how interventions, such as additional information, direct experience or positively portraying insects, could influence these factors.

## Figures and Tables

**Table 1:** Brambell’s Five Freedoms [28].

Five Freedoms	How to Achieve Them
Freedom from Hunger and Thirst	by ready access to fresh water and a diet to maintain full health and vigour.
Freedom from Discomfort	by providing an appropriate environment including shelter and a comfortable resting area
Freedom from Pain, Injury or Disease	by prevention or rapid diagnosis and treatment
Freedom to Express Normal Behavior	by providing sufficient space, proper facilities and company of the animal’s own kind
Freedom from Fear and Distress	by ensuring conditions and treatment which avoid mental suffering
